# Xenotransplantation of canine spermatogonial stem cells (cSSCs) regulated by FSH promotes spermatogenesis in infertile mice

**DOI:** 10.1186/s13287-019-1250-9

**Published:** 2019-05-20

**Authors:** Naira Caroline Godoy Pieri, Ana Carolina Furlanetto Mançanares, Aline Fernanda de Souza, Hugo Fernandes, Angela Maria Gonella Diaza, Fabiana Fernandes Bressan, Kelly Cristine Santos Roballo, Juliana Barbosa Casals, Mario Binelli, Carlos Eduardo Ambrósio, Daniele dos Santos Martins

**Affiliations:** 1 Department of Surgery, Faculty of Veterinary Medicine and Animal Sciences, University of São Paulo, Av. Prof. Dr. Orlando Marques de Paiva, 87, Sao Paulo, SP Brazil; 2 Department of Reproduction, Faculty of Veterinary Medicine and Animal Sciences, University of São Paulo, Av. Prof. Dr. Orlando Marques de Paiva, 87, Sao Paulo, SP Brazil; 3 Department of Animal Science, Faculty of Veterinary Science, Universidad de Concepción, Chillan, Chile; 4 Department of Veterinary Medicine, Faculty of Animal Sciences and Food Engineering, University of São Paulo, Av. Duque de Caxias Norte, 225, Pirassununga, SP Brazil; 5 North Florida Research and Education Center, Institute of Food and Agricultural Sciences, University of Florida, Marianna, FL USA; 6 School of Pharmacy at University of Wyoming, 1000 E. University Avenue, Laramie, 82071 USA; 7 Department of Animal Sciences, University of Florida, L.E. “Red” Larson Building, Bldg. 499, Room 122 C, Gainesville, FL 32611-0910 USA

**Keywords:** Transplant, Spermatogenesis, Hormone, Germ cells

## Abstract

**Background:**

Xenotransplantation of spermatogonial stem cells (SSCs) has become a popular topic in various research fields because manipulating these cells can provide insights into the mechanisms associated with germ cell lines and the entire spermatogenesis process; moreover, these cells can be used in several biotechnology applications. To achieve successful xenotransplantation, the in vitro microenvironment in which SSCs are cultured should be an ideal microenvironment for self-renewal and similar to the in vivo testicular microenvironment. The age of the donor, the correct spermatogenesis cycle, and the quality of the donor tissue are also important. Although cell culture-related factors, such as the in vitro supplementation of hormonal factors, are known to promote successful xenotransplantation in mice, little is known about the influence of these factors on SSCs in vitro or in vivo in other mammalian species, such as dogs (*Canis lupus familiaris*). In this context, the goals of this study were to test the effect of follicle-stimulating hormone (FSH) on canine spermatogonial stem cell (cSSC) cultures since this hormone is related to the glial cell-derived neurotrophic factor (GDNF) signaling pathway, which is responsible for the self-renewal and maintenance of these cells in vivo, and to investigate the microenvironment of the SSC culture after FSH supplementation. Additionally, in vivo analyses of transplanted FSH-supplemented cSSCs in the testes of infertile mice were performed to assess the capacity of cSSCs to develop, maintain, and restore spermatogenesis.

**Methods:**

SSCs from canine prepubertal testes (aged 3 months) were cultured in vitro in the presence of FSH (10 IU L^−1^). GFRA1 transcript expression was detected to confirm the spermatogonia population in culture and the effect of FSH on these cells. The protein and transcript levels of late germ cell markers (GFRA1, DAZL, STRA8, PLZF, and CD49f) and a pluripotency marker (OCT4) were detected at 72 and 120 h to confirm the cSSC phenotype. In vivo experiments were performed by transplanting GFP+ cSSCs into infertile mice, and a 10-week follow-up was performed. Histological and immunofluorescence analyses were performed to confirm the repopulation capacity after cSSC xenotransplantation in the testis.

**Results:**

Supplementation with FSH in cell culture increased the number of cSSCs positive for GFRA1*.* The cSSCs were also positive for the pluripotency and early germline marker OCT4 and the late germline markers PLZF, DAZL, C-kit, and GFRA-1. The in vivo experiments showed that the cSSCs xenotransplanted into infertile mouse testes were able to repopulate germline cells in the seminiferous tubules of mice.

**Conclusions:**

In conclusion, our results showed for the first time that the treatment of cSSC cultures with FSH can promote in vitro self-renewal, increase the population of germline cells, and possibly influence the success of spermatogenesis in infertile mice in vivo.

## Background

Spermatogonial stem cells (SSCs) are responsible for promoting the maintenance of spermatogenesis [1, 2]. Therefore, SSCs could be widely used in a number of recently developed and important biotechnologies, such as restoring fertility in injured animals, generating transgenic animals and preserving endangered species [3–5]. SSCs of different species have been used for xenotransplantation in several studies, and this technique is extremely desirable because it can provide insights to reveal the entire spermatogenesis process and improve germline repopulation [4, 6–11]. However, to optimize xenotransplantation results, a number of important points need to be addressed simultaneously, such as the ideal microenvironment for germ cell culture, the age of the donor, the correct spermatogenesis cycle, and the quality of the donor tissue [11–14].

Special precautions are required to maintain SSCs in vitro in specific media with supplements, such as FSH. In males, FSH plays an important role in spermatogenesis because it can promote Sertoli cell proliferation, increase cell differentiation, support many aspects of sperm cell maturation, and play a role in the mitotic activity of spermatogonia [15]. In addition, FSH has been used to stimulate the proliferation of spermatogonia via the GDNF-GFRA1 pathway and control the germline stem cell niche to create a suitable environment that promotes the differentiation and proliferation of these cells [16, 17].

FSH can increase GDNF expression when added to primary cultures of mouse Sertoli cells [18], and when FSH was used for 7 days or more in mouse spermatogonia cells cultured in vitro, a significant induction of the expansion of associated spermatogonia with the appearance of meiotic cells was demonstrated [19].

Although cell culture-related factors (hormones, age, in vitro supplementation, etc.) were reported to promote the success of xenotransplantation in mice, little is known about the influence of these factors in other mammalian species, such as dogs (*Canis lupus familiaris*). In dogs, few xenotransplantation experiments using germ cells have been performed with success [7, 10, 11, 20], and none of these studies have tested the influence of FSH or other types of gonadotropins on cSSCs in vitro or in vivo.

One of the first studies [7] using cSSCs reported the importance of dogs as experimental models. Dogs are considered important biomedical models for transplant studies because they present more than 400 hereditary diseases that are similar to human diseases [21].

Thus, the purpose of this study was to evaluate the behavior and profile of cSSCs in vitro when cultured with FSH and to analyze FSH-supplemented cSSCs after transplantation in the testes of infertile mice to assess the capacity of cSSCs to develop, maintain, and restore spermatogenesis.

## Methods

All protocols applied in this study and procedures involving animals were conducted in accordance with the Ethics Committee of the Veterinary Medicine School at the University of São Paulo, Brazil (2549/2012).

### Collection of canine testes

Canine testes were obtained from 3-month-old healthy prepubertal mongrel dogs (*N* = 5) from a dog population control campaign conducted in Pirassununga, Sao Paulo, Brazil. Orchiectomy was performed by a veterinary surgeon. All animals were anesthetized with 0.05 mg/kg acepromazine + 2 mg/kg meperidine ± intramuscular 5 mg/kg propofol ± intravenous and inhaled anesthesia with 1.5 ± 2.5% isoflurane ± continued throughout the surgery. The post-surgical treatment was composed of antibacterial therapy (20 ± 30 mg/kg cephalexin ± oral ± once every 12 h), analgesic treatment (1 ± 4 mg/kg tramadol ± intramuscular ± once every 12 h), anti-inflammatory treatment (0.1 ± 0.2 mg/kg meloxicam ± oral ± once every 24 h), and local care (rifamycin).

Tissues were immediately washed in phosphate-buffered saline (PBS) to remove cell debris and potential contaminants. The testis capsule was then removed and mechanically dissociated, and testis cells were isolated and cultured, followed by flow cytometric and RT-qPCR analyses.

### Germ cell in vitro culture

Testes tissue samples (*N* = 5) were digested and cultured as described by Pieri and collaborators [22]. First, germ cells and somatic cells were cultured in Dulbecco’s modified Eagle medium/F12 (Cat#BR-30004-05, LGC, Cotia, Brazil) supplemented with 10% fetal bovine serum, 2 mM l-glutamine (Cat#12657029, Cat#25030-081, respectively; Gibco/Invitrogen, Carlsbad, CA, USA), 100 U/mL penicillin/streptomycin, 50 μL of pyruvate (Cat#15140-122, Cat#11360-070, respectively; Thermo Fisher Scientific, Waltham, MA, USA), and 100 U/L amphotericin B (Cat#A2942; Sigma-Aldrich Corp., St. Louis, MO, USA). Second, spermatogonia were purified by using Percoll® (Cat#P1644, Sigma-Aldrich Corp., St. Louis, MO, USA), and cells were collected from the 27–35% fractions. Third, the cells were resuspended in the culture medium, plated and incubated at 38 °C under 5% CO_2_ [22].

Finally, the cSSCs were co-cultured with Sertoli cells and treated with 10 IU L^−1^ FSH (Cat#4021, Sigma-Aldrich Corp., St. Louis, MO, USA) [23]. These cells were trypsinized and collected after 72 and 120 h of in vitro treatment and were separated into two groups: one supplemented with FSH and another without FSH (control).

### Flow cytometry analysis of cSSCs supplemented with FSH

cSSCs supplemented or not with FSH were evaluated by flow cytometry after 72 and 120 h in culture [22] for germ cell marker analysis. For this purpose, 8 × 10^4^ cSSCs were incubated with the primary antibodies GFRA1 and PLZF (1:50, sc10716 and sc22839, respectively; Santa Cruz Biotechnology, Dallas, TX, USA); DAZL and C-kit (1:100, 1:50, ab34139, ab5506, respectively; Abcam, Cambridge, UK); and STRA8, Alpha 6 integrin (CD49f), and OCT4 (1:100, ab34139, ab20142, and ab18976, respectively; Abcam, Cambridge, UK) with 1% BSA for 1 h at room temperature. The secondary antibody used was goat anti-rabbit (H + L) Alexa 488 (1:300; Cat#A-11034, Invitrogen, Carlsbad, CA, USA). A negative control was performed with isotype-matched rabbit polyclonal IgG (ab27478, Abcam, Cambridge, UK). The cells were evaluated in a FACSCalibur cytometer (BD Biosciences, San Jose, CA, USA) [22].

The first statistical analysis was an assessment of the percentage of GFRA1-positive cells in cSSCs with/without FSH supplementation at 72 and 120 h in culture by two-way analysis of variance (ANOVA, *p* ≤ 0.05). The second statistical analysis was an evaluation of the phenotype of the cSSCs. The percentages of GFRA1-, PLZF-, OCT4-, DAZL-, CD49f- and C-kit-positive cells in cSSCs with/without FSH supplementation were determined before xenotransplantation into mice using mean values and paired *t* tests (*p* < 0.05). All statistical analyses were performed using GraphPad Prism v.6 software.

### Generation of GFP-positive cSSCs

Approximately 10^6^ cSSCs were transduced with the lentivirus vector FUGW (vector encoding the GFP gene) as described previously [24–26]. Transduction was performed in a 24-well dish, and each well received the lentiviral supernatant with 8 μg/ml of polybrene. Twenty-four hours after transduction, GFP-positive cells were sorted using a FACSCalibur cytometer (BD Biosciences, San Jose, CA, USA) and then re-cultured for the next experiments. The cell cultures were maintained in 24-well plates and transplanted into recipient mouse testes. Flow cytometry was used to evaluate the efficiency of transduction (BD FACSAria™, BD Biosciences, San Jose, CA, USA) along with BD FACSDiva™ software (BD Biosciences, San Jose, CA, USA).

### Analysis of GFRA1 gene expression by RT-qPCR

Total RNA was isolated from the cSSCs cultured with or without FSH treatment after 72 and 120 h in culture using the TRIzol reagent (Life Technologies, Carlsbad, CA, USA) according to the manufacturer’s recommended protocol. The concentration and purity of the total RNA were estimated using a spectrophotometer (NanoDrop, Thermo Fisher Scientific, Waltham, MA, USA). cDNA synthesis was performed using a High-Capacity Reverse Transcription kit (Applied Biosystems, CA, USA) following the manufacturer’s protocol.

The primer sequences for the germline marker GFRA1 (forward, 5′-GCAAGTGGAGCACATTCCCA-3; reverse, 5′-CAGACCTCGTTGGACATGCT-3′) and the housekeeping gene 18S (forward, 5′-CCTGCGGCTTAATTTGACTG-3′; reverse, 5′-CTGTCAATCCTGTCCGTGTC-3′) were designed using primer 3. The PCR amplification of the selected germinative gene was performed using a StepOne Plus (Applied Biosystems Inc., Foster City, CA, USA) instrument with SYBR Green Master Mix (Applied Biosystems Inc., Foster City, CA, USA). RT-qPCR was performed as follows: one pre-cycle at 95 °C for 10 min; 40 cycles of 95 °C for 15 min, 65 °C for 1 min, and 72 °C for 30 s; and a final step at 72 °C for 2 min. All reactions were performed with biological and technical replicates (*N* = 3). The transcript levels were determined by RT-qPCR and analyzed with LinReg PCR software (Version 2015.0). The cycle threshold (Ct) values of target gene expression were normalized by using Ct values of 18S (endogenous gene) for each sample. Following normalization, the fold change was calculated by using the Pfaffl equation [27]. Adult canine testes were used as a positive control.

For statistical analyses, analysis of variance (ANOVA, *p* < 0.05) was performed first, followed by an analysis of the difference between the measured gene expression, and group means were evaluated by Tukey’s test (*p* < 0.05); all tests were performed using GraphPad Prism v.6.

### Xenotransplantation of SSCs

#### Chemical treatment—immunosuppression of mice

Busulfan (1,4-butanediol dimethanesulfonate) (Cat#B2635, Sigma-Aldrich Corp., St. Louis, MO, USA) was diluted in dimethyl sulfoxide (DMSO; Sigma-Aldrich Corp., St. Louis, MO, USA) solution at a final concentration of 3.8–4 mg/mL to induce germ cell loss [28]. A drug dose of 45 mg/kg of body weight was administered intraperitoneally to each C57BL/6 mouse (5–6 weeks old, *N* = 6) [28]. Control animals received only DMSO. All animals were maintained at a temperature of 22+/− 2 °C with food and water available ad libitum*.* The mice were evaluated after 6 weeks of drug administration. An analysis of reproductive parameters (testicular mass, tubular diameter, and population of germ cells) and an immunohistochemical analysis of the spermatogonium marker promyelocytic leukemia zinc finger protein (PLZF) were performed to confirm that the mice were infertile [22].

#### In vivo experimental design—xenotransplantation of canine SSCs in mouse testes

All recipient mice (6 weeks old, *N* = 9) were treated with busulfan and divided into three groups: G1—mice that received cSSCs supplemented with FSH (*N* = 3); G2—mice that received canine SSCs without FSH (*N* = 3); and G3—surgery control group receiving only saline injection (*N* = 3). Before transplantation, cSSCs were washed three times in PBS and resuspended at a concentration of 1 × 10^5^ cells/10 μL, which resulted in up to 80% filling of the recipient mouse seminiferous tubules. Trypan blue (0.001%) (Cat #15250-061, Invitrogen, Carlsbad, CA, USA) was used to track and guarantee injection inside the seminiferous tubules.

Cell injection was performed via the deferent duct and rete testis according to a protocol described previously [28–30]. The animals were anesthetized with ketamine (50 mg/kg) and xylazine (15 mg/kg), and the testicles were externalized via an incision in the abdominal wall of 1 to 1.5 cm and a 2 to 3 mm incision in the tunica dartos muscle. Using a stereomicroscope (Nikon, Chiyoda, Tokyo, Japan) and an Ultra-Fine™ syringe (0.30 mm; 30G, BD Becton Dickinson, East Rutherford, New Jersey, USA), cSSCs together with 0.001% trypan blue were injected into the efferent duct of each testis. After filling the tubules, the testes were returned to the abdominal cavity and the muscle layer and skin were sutured with 0–6 mm wire (Silkam®, B. Braun Melsungen, Melsungen, Germany) (Fig. 4A).

At 10 weeks after transplantation, the mice were evaluated for the presence and localization of donor-derived cSSCs in their seminiferous tubules by fluorescence analysis under light microscopy (KS400; Carl Zeiss, Göttingen, Germany) using AxioVision v.4.6 software.

#### Analyses of recipient testis—histology and fluorescence

The animals were euthanized in carbon dioxide (CO_2_) in accordance with ethical standards. Sample testes were fixed in freezing medium (Tissue-Tek O.C.T. Compound, Cat#Saku-4583, Sakura American, Sakura Finetek, USA) and stored at − 80 °C. GFP+ cSSCs were identified and counted [31]. For precise and comparative analyses, the total numbers of cells positive and negative for GFP were counted, and the percentage of GFP+ cSSCs in the tubules of three sections of three random fields observed under a × 200 objective by fluorescence microscopy (KS400; Zeiss, Göttingen, Germany) was calculated using AxioVision v.4.6 software (Carl Zeiss, Göttingen, Germany) [32–35]. Statistical analysis of the effect of FSH in the recipient mice after transplantation of cSSCs was performed using the *t*-means and nonparametric (Mann-Whitney, *p* < 0.05) statistical tests.

#### Molecular detection of GFP+ cSSCs

To detect the presence of GFP+ cSSCs in the recipient mice after transplantation, the genomic DNA from the epididymis was isolated by using a QIAmp tissue kit (Cat #69509, Qiagen Inc., Santa Clarita, CA, USA). DNA from mouse testes that received GFP cSSCs was amplified by qualitative PCR using primers specific for the canine α-satellite DNA [7]. The PCR amplification of α-satellite DNA was conducted with 1 μM concentrations of the primers (5′-AACCTTTCCCTGCCACTAAC-3′ and 5′-CTCACCCTCAGTCCTTCACA-3′) [36]. The PCR conditions consisted of initial denaturation at 95 °C for 1 min, followed by 30 cycles of denaturation at 94 °C for 1 min, annealing at 64 °C for 1 min, and elongation at 72 °C for 1 min. The PCR products (324 bp) were visualized in a 1.5% agarose gel. Mouse genomic DNA was used as a negative control, and the positive control was genomic DNA from a dog. All samples from non-injected mouse testes (negative control) were subjected to PCR with primers specific for the mouse SRY gene (5′-AGATATCATGTGGCTGTAGG-3′ and 5′-CTAACAGCTGACATCACTG-3′) [37].

## Results

### Isolation of cSSCs and influence of FSH in vitro

**Fig. 1:**
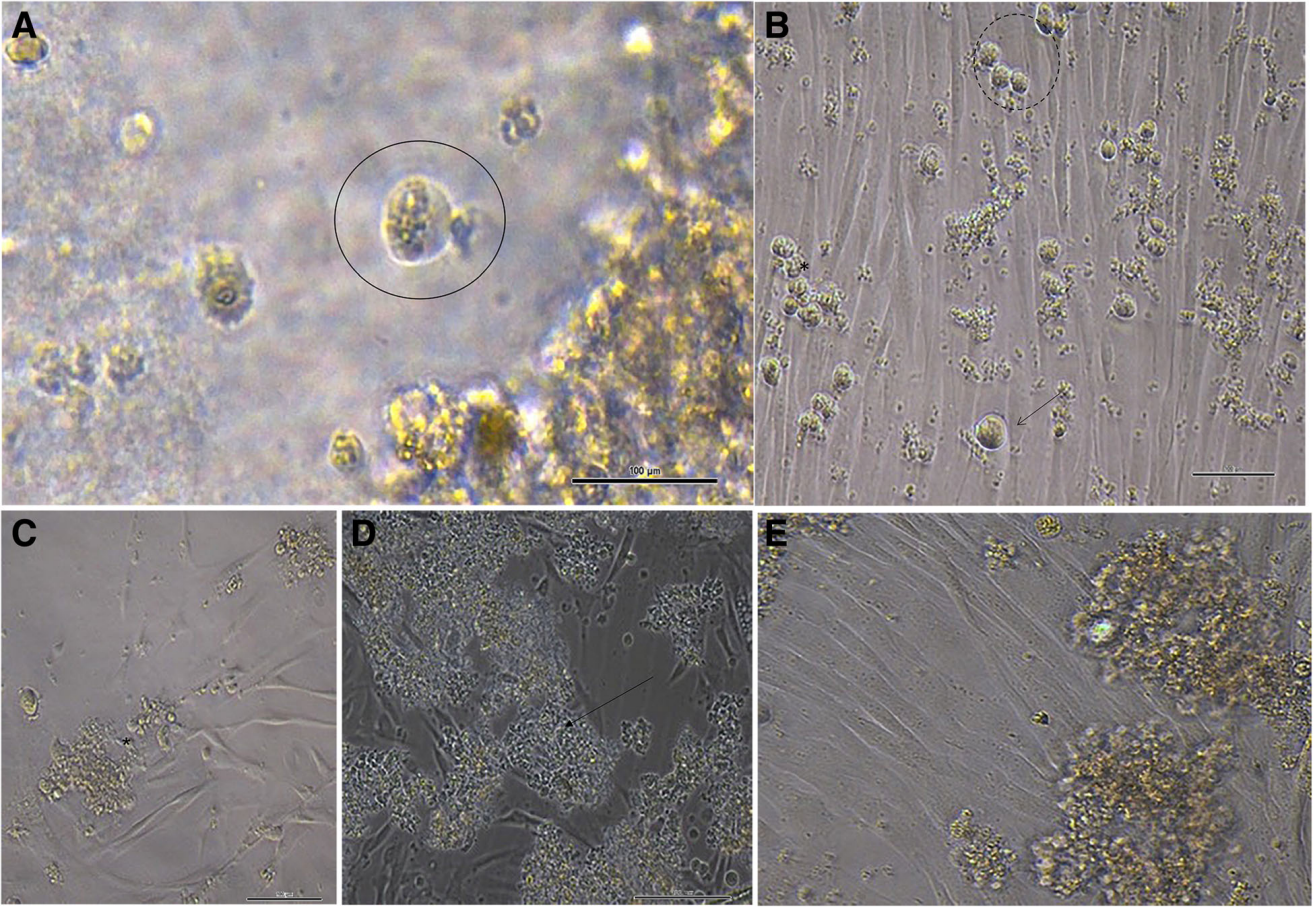
**a** Typical morphology of spermatogonia: rounded or oval cells and spiral nucleus related to the cytoplasm (circle). **b**, **c** Microscopic appearance of the formation of germ cell clumps (arrow) in small groups of cells (dotted circle) and continuous proliferation of clump-forming cells (asterisk) (2 days and 15 days after in vitro culture of cSSCs). **d**, **e** Formation of clumps by cells in suspension (arrow) among Sertoli cells (asterisk). (Scale bar = 100 μm)

**Fig. 2:**
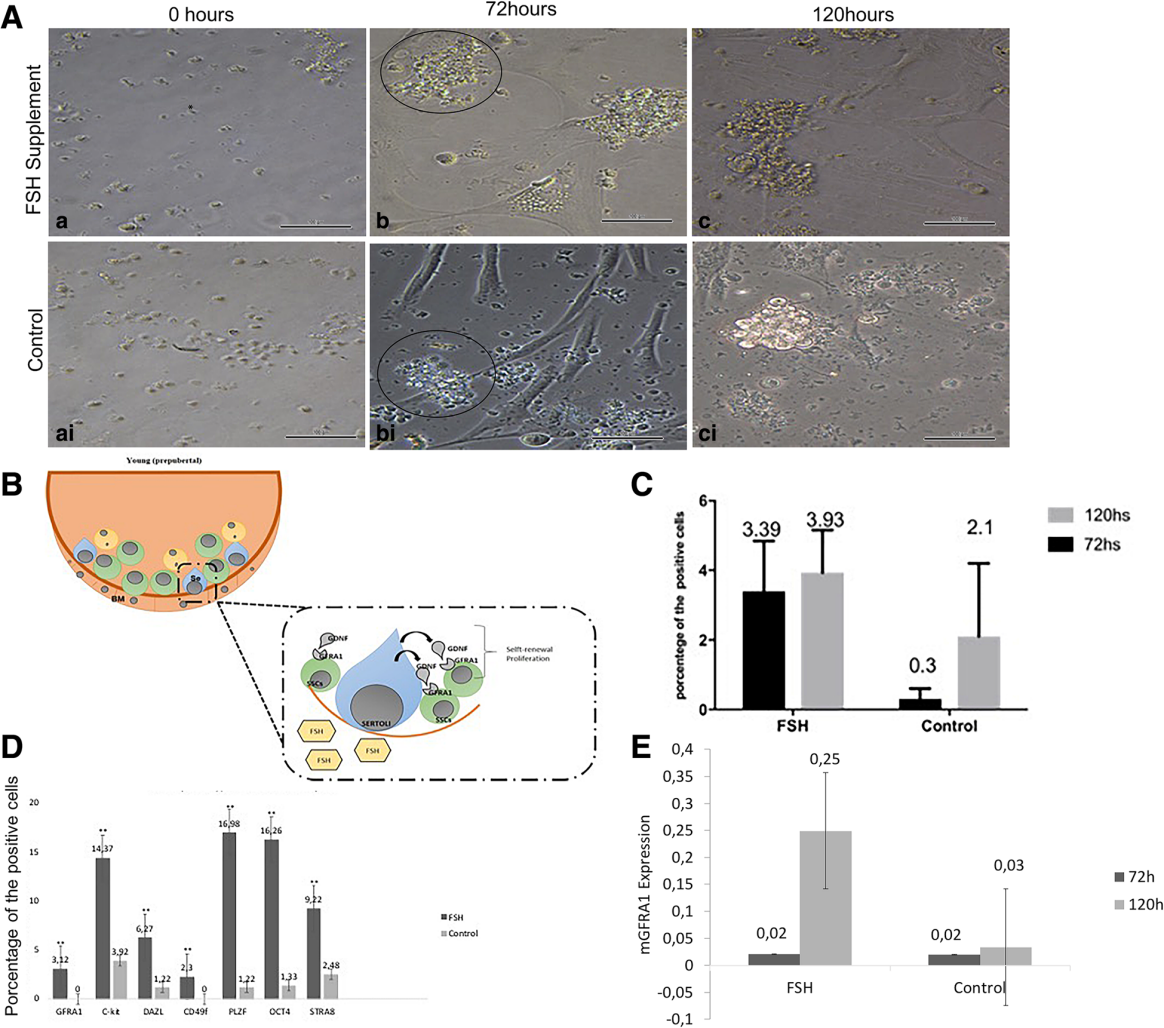
**a** Canine SSCs supplemented with FSH in vitro and the control group were evaluated at 0, 72 and 120 h. **a**–**c** cSSCs (asterisk) supplemented with FSH in culture showed an increase in the number of cells and formed germ cell clumps after 72 h (arrow). These cells did not show a difference in morphological features compared with the control group up to at least 120 h. **ai**–**ci** These cells formed clumps after 72 h in vitro (scale bar = 100 μm). **b** Illustration demonstrating the influence of FSH on the cSSCs. Specific receptors in the testes, namely, follicle-stimulating hormone receptor (FSHR), are bound in Sertoli cells and Leydig cells. Under the influence of FSH, Sertoli cells release GDNF, and this paracrine factor then binds to the GFRA1/Ret protein localized in the membrane of SSCs and initiates the self-renewal process in these cells. **c** Flow cytometric analysis of the percentage of GFRA1positive cSSCs after 72 and 120 h of treatment with FSH supplementation and the control (*p* ≤ 0.05). **d** Graph showing the phenotypes of the cSSCs for germ cell markers (GFRA1, c-kit, DAZL, CD49f, PLZF, OCT4, STRA8) before xenotransplantation of the cSSCs (*p* < 0.05). **e** Gene expression of mGFRA1 evaluated at 72 and 120 h after FSH supplementation and in the control (*p* < 0.05)

The in vitro cultures of cSSCs showed a heterogeneous cell population, including spermatogonia and somatic cells. The cSSCs showed typical morphological features: a round and oval shape with a high nucleolus/cytoplasm ratio without any changes during in vitro culture (Fig. 1). These cells appeared to be isolated or formed germ cell clumps after 2 days in culture (Fig. 1b). The germ cell clumps continuously proliferated for 15 days (Fig. 1c–e). Importantly, the morphological features of cSSCs supplemented with FSH were analyzed and compared with that of the control group (Fig. 2Aa–ci).

The flow cytometry assay showed an increase in GFRA1 protein levels (3.66 ± 0.9765) in cSSCs over time (72 or 120 h) in the group of cSSCs supplemented with FSH compared to the control group (1.2 ± 1.044) (*p* < 0.05) (Fig. 2C). We also characterized the cSSCs regarding the presence of pluripotent and germ cell markers. The flow cytometry assay showed that in cSSCs supplemented with FSH, more than 15% of the cells evaluated were positive for OCT4 (16.26%), C-kit (14.37%), and PLZF (16.98%), whereas less than 5% of the cells were positive for GFRA1 (3.12%) and DAZL (6.27%). In contrast, in the cells from the control group, only 1.33% OCT4+, 1.22% PLZF+, 0% GFRA1+, 3.92% C-kit+, and 1.22% DAZL+ cells were observed. The statistical analysis showed a significant difference (*p* > 0.05) (Fig. 2D).

RT-qPCR demonstrated that the *GFRA1* gene was abundant in the cSSCs supplemented with FSH. Importantly, there was a 5-fold increase in the transcription of *GFRA1* in these cells after 120 h (Fig. 2E)*.* These results suggest that FSH supplementation together with Sertoli cells can increase germline and stem cSSC populations and promote proliferation and self-renewal in vitro via the GDNF signaling pathway (Fig. 2B).

**Fig. 3:**
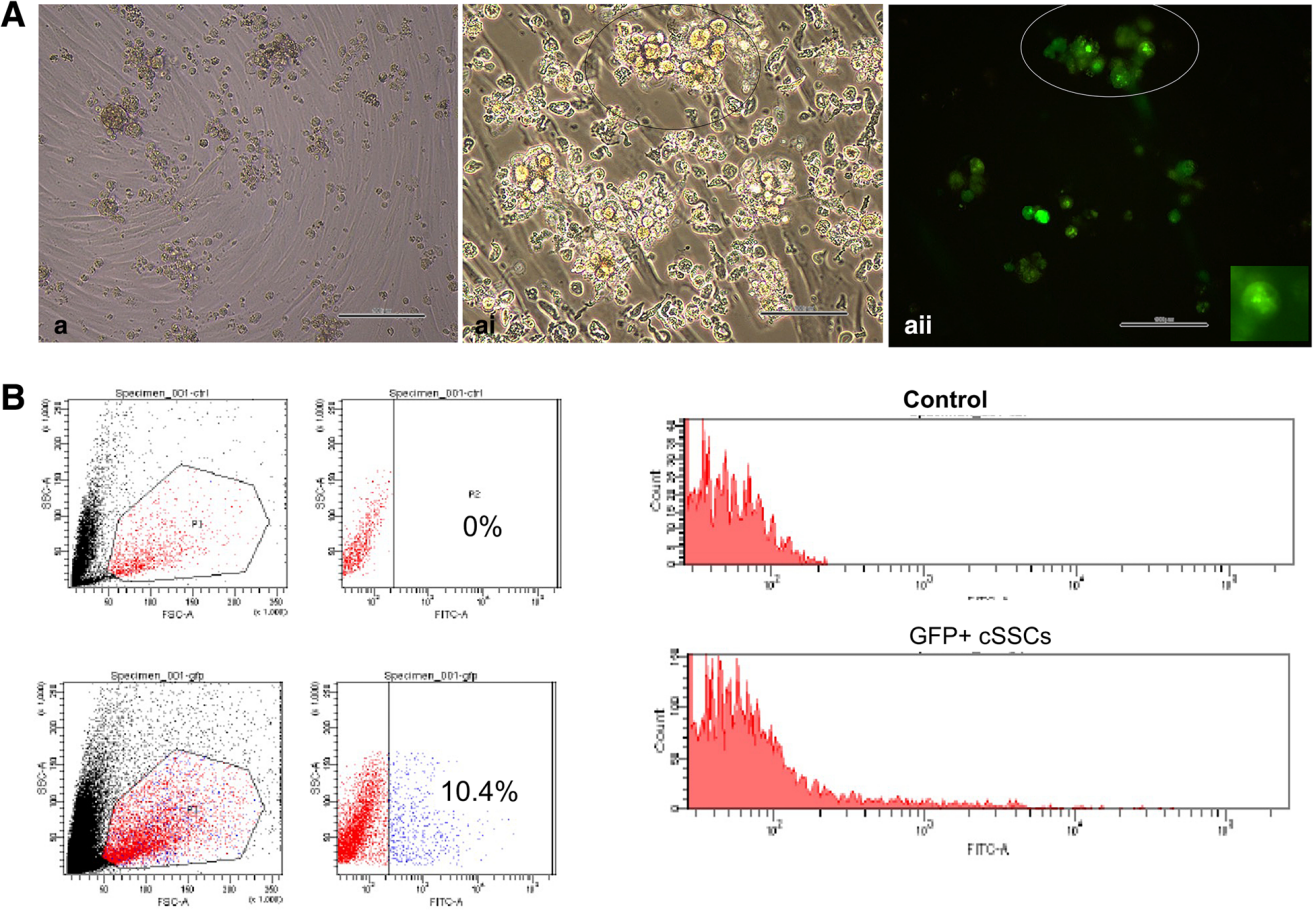
Flow cytometric and fluorescence analysis of cSSCs. **a**–**ai** GFP+ cSSCs appear as isolated cells or form germ cell clumps (circle). **aii** Fluorescence analysis of cSSCs showing GFP positivity after transduction (circle). **B** Flow cytometry was used to evaluate the efficiency rate of the transduction of cSSCs with GFP compared with the control group. The control group (histogram) was GFP negative. The histogram shows that in the treated group, only 10.4% of the cSSC population was GFP+. (Scale bar = 100 μm)

When cSSCs were transduced with GFP, the flow cytometric analysis showed that 10.4% of the cells expressed GFP. When the control group (not transduced) was analyzed, GFP+ cSSCs were not observed (Fig. 3).

### Xenotransplantation of canine spermatogonial stem cells (cSSCs)

**Fig. 4:**
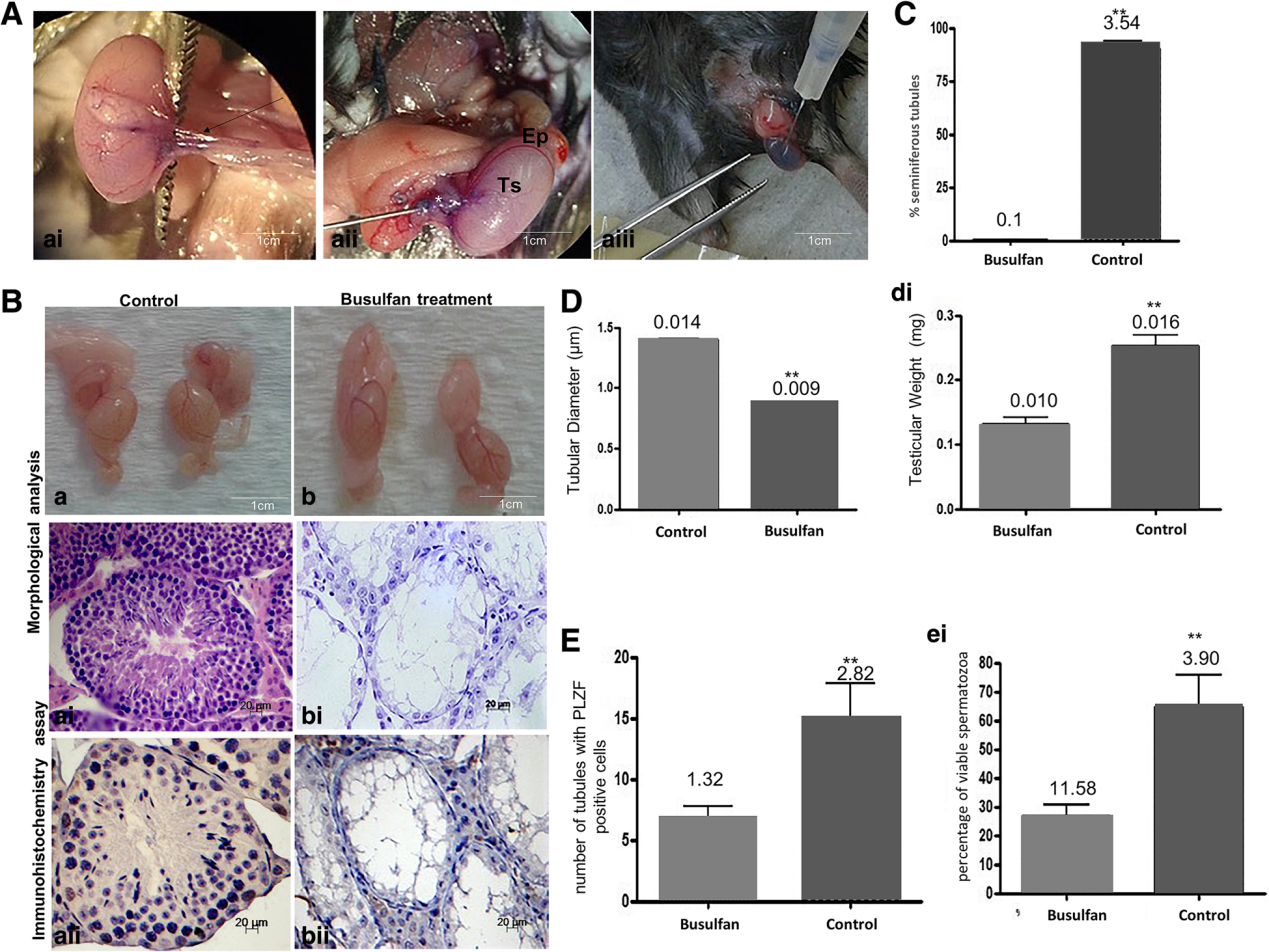
**a**–**e** Effect of treatment with busulfan in mouse testes after 6 weeks, before cSSC xenotransplantation. **a** Technique for the transplantation of cSSCs into the mouse testes. **ai**–**aiii** Insulin needle inserted into the efferent duct (asterisk) located the testicular artery cranially (arrow) between the tail of the epididymis (Ep) and testis (Ts) to help delineate the ducts and the testicular network, and then, all seminiferous tubules were filled with a small amount of blue dye solution (Trypan Blue). **b** Macroscopic and microscopic analyses of the mouse testes before xenotransplantation: control and treated (busulfan) groups (scale bar = 1 cm, 20 μm). **ai**–**aii** Control group, spermatogenesis was normal. It was possible to observe complete spermatogenesis (spermatogonia, spermatocytes, and spermatozoa) in the mouse seminiferous tubules. **b**–**bii** Treated group did not exhibit germ cells in the germinative epithelium. In the seminiferous tubules, only Sertoli cells (Se) were present. **c** Graphic showing the statistical analysis of the mean number of seminiferous tubules exhibiting normal spermatogenesis in the control and treated groups. **d** Graphic showing the changes in tubular diameter and testicular weight in the control and treatment groups. **e** Graphic showing the number of cells that expressed the PLZF marker. **ei** Number of spermatozoa after busulfan treatment was performed to confirm busulfan efficiency. **Significant difference (*p* < 0.05). (Scale bar = 20 μm)

Before xenotransplantation, the mice were treated with busulfan. After 6 weeks, germ cell-depleted seminiferous tubules were observed in the busulfan-treated mice (Fig. 4B). No physiological changes in these animals were observed during the experiment. Importantly, decreases in testicular mass, tubular diameter, and the germ cell population and a reduction in seminiferous tubules were observed in the treated mice (81.67%) (Fig. 4C–E). Immunohistochemical analysis of PLZF revealed its expression in spermatogonia, and no difference in the state or number of spermatozoa after busulfan treatment, which was performed to confirm the efficiency of busulfan (Fig. 4Baii-bii and E). This analysis also showed that after 6 weeks, there were reduced numbers of spermatogonia in the seminiferous tubules and viable spermatozoa and only Sertoli cells were present in the basement membrane (Fig. 4Bai-bi-Eei). The results showed that the mice were infertile and could be used in the xenotransplantation experiment.

**Fig. 5:**
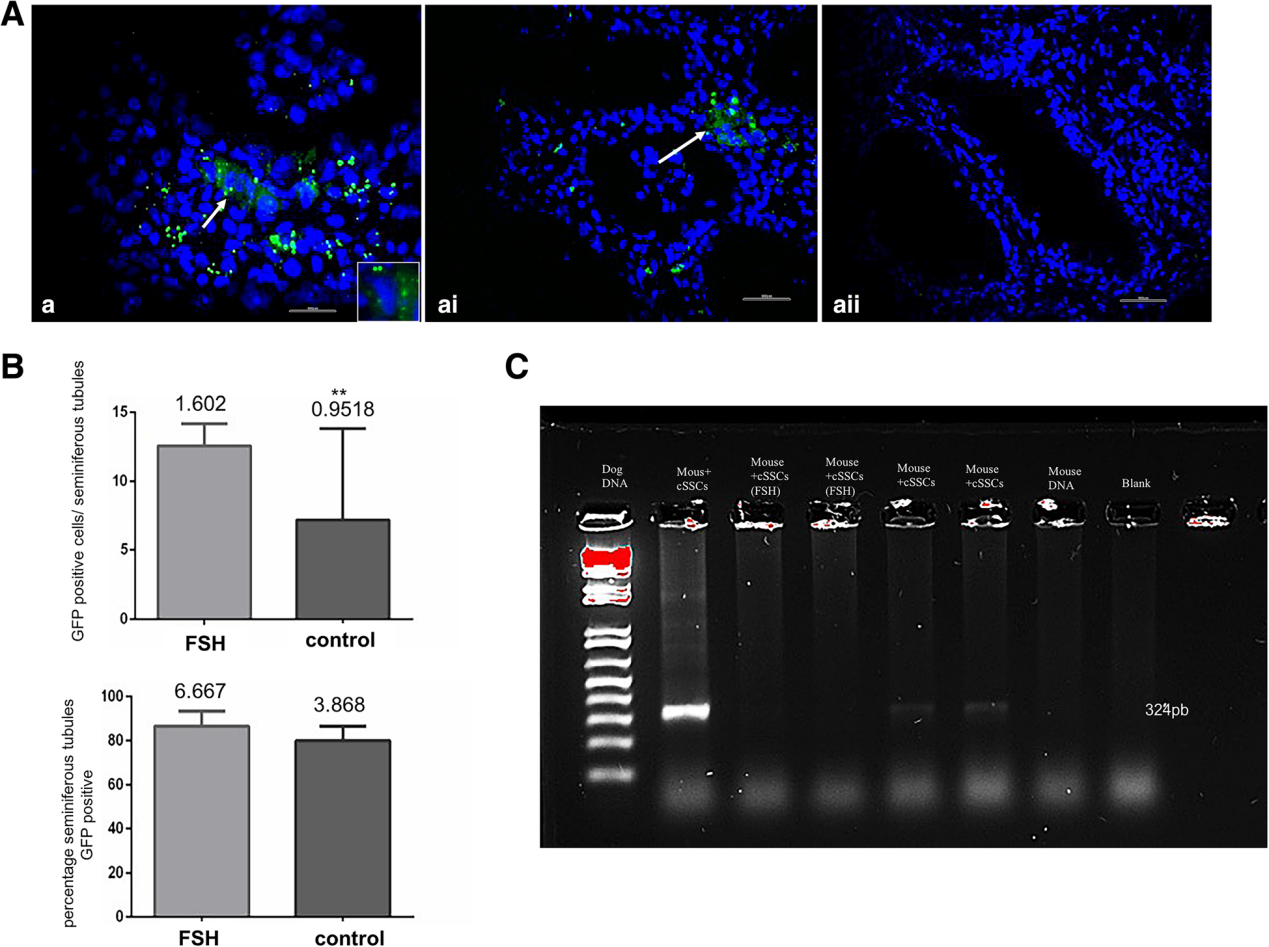
Analyses of mouse testes after xenotransplantation of cSSCs. **a** Histological analysis of the mouse testis at 10 weeks (70 days) after xenotransplantation. **a**–**ai** In mice that received cSSCs treated with FSH in vitro, GFP-positive cells were observed in the seminiferous tubules (arrow). **aii** In the testes of mice that received control cSSCs (not treated with FSH), GFP-positive cSSCs were not observed in the seminiferous tubules (scale bar = 100 m**)**. **b** Graphic showing the number of GFP-positive cSSCs in the seminiferous tubules 10 weeks after transplantation in the control and treated groups. The second graphic shows the percentage of seminiferous tubules exhibiting GFP-positive cSSCs compared with those of the control and treated groups. **Significant difference. Unpaired *t* test (*p* ≤ 0.05). **c** Polymerase chain reaction (PCR) analysis of mouse testes at 10 weeks after the transplantation of cSSCs. Evaluation of the presence of dog DNA in mouse testes. Positive control (canine fibroblasts), negative control from cells (mouse fibroblasts), and the blank control

After xenotransplantation of the cSSCs into mouse testes, an analysis of the seminiferous tubules of the mice by fluorescence microscopy showed that canine GFP+ cSSCs were present in the basal layer of tubules. After 10 weeks, GFP+ cSSCs were detected as single cells or small clusters among the interstitial cells of mouse testes (Fig. 5A).

Specifically, 9.8% of the transplanted cells were GFP+ (10^5^ cells/testis), whereas 90.2% of cells present in the testis were GFP− (total of 2760 cells counted). An average of 2.089 ± 0.28 cells/tubule was found for GFP+ cSSC colonies in the seminiferous tubules in the mice that received cSSCs supplemented with FSH. When the percentage of GFP+ cells was evaluated after xenotransplantation in the tubules, we observed approximately 86.63% of GFP+ cells, and the mean number of these cells per tubule was 12.57 ± 1.60. However, in the mice that received the cells without FSH treatment, 1.7% of the cells were GFP+ and 98.3% were negative (total of 2746 cells counted). In the recipient mice after transplantation, 80% of the tubules had GFP+ cSSCs, and the mean values for GFP+ cSSCs were 7.16 ± 0.95 cells/tubule and 1.22 ± 0.17 GFP+ colonies/tubule. Statistical analyses showed a difference in the mean numbers of positive cells and colonies between the groups (*p* ≤ 0.05). However, the percentage of positive cells in the tubules did not differ after transplantation (Fig. 5B). Therefore, these data demonstrated that the mice that received cSSCs supplemented with FSH in vitro presented improved parameters regarding the numbers of cells and colonies and the percentage of seminiferous tubules positive for GFP+ cSSCs at 10 weeks (70 days) after transplantation (Fig. 5).

### PCR detection of xenotransplantation in the epididymis

PCR analysis showed that only tissue collected from recipient mice that received cSSCs supplemented with FSH exhibited canine DNA in the epididymis (Fig. 5C).

## Discussion

Studies have demonstrated that the induction and maintenance of spermatogenesis are dependent on various reproductive hormonal pathways, including the FSH, testosterone (T), and luteinizing hormone (LH) pathways [38–40]. The secretion of these hormones relies on the hypothalamic-pituitary-testicular axis, which maintains testicular function and fertility [41]. The gonadotropin FSH plays a crucial role in spermatogenesis by activating the proliferation of Sertoli cells, influencing the mitotic activity of spermatogonia, and increasing cell differentiation [15, 38]. Although it is well established in the literature that gonadotropin signaling plays a pivotal role in the spermatogenesis of vertebrates, exactly how FSH may regulate the self-renewal of spermatogonia in canines remains inconclusive [42]. Here, we studied the influence of FSH in cSSCs both in vitro and in vivo. Importantly, in our in vivo xenotransplantation experiment, it was proved that cSSCs supplemented with FSH can restore spermatogenesis the recipient animal.

In this study, the cSSCs appeared isolated or formed germ cell clumps similar to mouse SSC cultures [15]. Another report involving mouse spermatogonia co-cultured with Sertoli cells and supplemented with FSH showed that the spermatogonia maintained the characteristics of undifferentiated germ cells [42]. Similarly, the cSSCs evaluated in this study did not undergo morphological changes in the presence of FSH.

Another study in which FSH supplementation was performed in mouse SSCs co-cultured with Sertoli cells showed that *GDNF* mRNA levels were increased by FSH and that the levels of mRNAs associated with germ cells, such as *c-Kit*, *Nanog*, and *PLZF*, did not change with culture time [43]. In our study, cSSC cultures were supplemented with FSH in vitro for up to 120 h. The *GFRA1* mRNA level changed with time compared to untreated cells. We observed that the gonadotrophin FSH and the co-culture of cSSCs with Sertoli cells could influence the self-renewal process by activating the GDNF-GFRA1 signaling pathway in these cells.

Using mice as a model, SSCs were co-cultured with Sertoli cells in serum-free medium and 20 ng/mL FSH. When FSH was added to the cultures, a significant increase in SSCs was observed at 7 and 14 days compared with control cells according to the total number of spermatogonia in the cultures evaluated by flow cytometry [15]. Herein, we cultured cSSCs with FSH and observed a similar influence of FSH on cultured cells. FSH treatment promoted an increase in the number of GFRA1-positive cells, which may have been related to FSH leading Sertoli cells to express glial cell line-derived neurotrophic factor (GDNF) of the alpha 1 receptor family (GFRA1) [44] because this factor promotes signaling in response to Sertoli cells that are targets of FSH in the testis and connects GFRA1 to spermatogonia [45, 46]. However, the specific mechanisms of FSH in spermatogonia cells are still unclear [47]. In fact, the observed increase in GFRA1 indicates that Sertoli cells produce mitogenic factors that control the effect of FSH on spermatogonia [48, 49].

SSCs from domestic animals, such as dogs, pigs, and rabbits, have been transplanted into the seminiferous tubules of germ cell-depleted infertile mice, although few of these experiments were able to restore spermatogenesis [4, 7, 12, 50, 51]. Our investigation showed that the xenotransplantation of cSSCs previously supplemented with the gonadotrophin FSH in vitro can lead to a higher rate of colony formation and successful colonization of the seminiferous tubules of recipient mice, although spermatogenesis was not completed [52]. Therefore, we conclude that the successful restoration of spermatogenesis in mice by cSSCs can be influenced by other factors associated with hormonal systems and age.

Importantly, gonadotrophins are among the factors that may influence the success of xenotransplantation in mammals [12]. In a previous study performed using dog testicular xenografts in FSH- and LH-treated mice, at 4 months after cell transplantation, mice that received cells from immature and young dogs exhibited a higher rate of recovery of spermatogenesis, graft weight, the vesicular gland index, and tubule diameter [53]. Although we used the same model, we observed a better proliferation rate in the seminiferous tubules in mice that received prepubertal cSSCs supplemented with FSH in vitro.

When the xenotransplantation assay between dogs and mice was performed previously [7] to demonstrate the efficiency of the technique and the model, the authors used a PCR-based method and detected one dog cell among 106 mouse cells analyzed. In our study, we performed the same technique for the detection of canine DNA and obtained positive results in mice, and one of these mice received germ cells treated with FSH in vitro.

Our research showed that cSSCs supplemented with FSH remained in the testes of mice treated with busulfan for long periods, which may influence the efficiency rate in the restoration of spermatogenesis. According to the literature, xenotransplantation of domestic animal tissues/cells in the testes is not possible because of the [14, 54] difference in the body temperature (~ 36–37.5 °C) of nude mice compared with the canine testis (33–35), which may cause germ cell depletion in the reconstructed germ cell testes following xenotransplantation. Other problems that can influence the restoration of spermatogenesis are age, environment, hormones, and the niche of SSCs [12].

## Conclusions

In conclusion, supplementation with FSH increases the number of cSSCs in culture, suggesting that FSH can promote the proliferation and increase the self-renewal rate of these cells. Additionally, cSSCs supplemented with FSH were able to colonize mouse seminiferous tubules in vivo and remained in the basement membrane for up to 10 weeks after transplantation, although these animals are phylogenetically distant and exhibited a variety of ages. Thus, the results indicate that successful xenotransplantation is possible between cSSCs and mice and demonstrate that this is a viable model that offers new insights for the treatment of infertility and understanding the mechanisms of spermatogenesis in domestic animals.

## Abbreviations

C-kit: Tyrosine-protein kinase kit
cSSCs: Canine spermatogonial stem cells
DAZL: Deleted in azoospermia-like
FSH: Follicle-stimulating hormone (FSH)
GDNF: Glial cell line-derived neurotrophic factor
GFP: Green fluorescent protein
GFRA1: GDNF family receptor alpha-1
LH: Luteinizing hormone
OCT4: Octamer-binding transcription factor 4
PBS: Phosphate-buffered saline
PLZF: Promyelocytic leukemia zinc finger
SSCs: Spermatogonial stem cells
STRA8: Stimulated by retinoic acid 8
